# Evidence-Based Integrated Analysis of Environmental Hazards in Southern Bolivia

**DOI:** 10.3390/ijerph16122107

**Published:** 2019-06-14

**Authors:** Francesco Cantini, Giulio Castelli, Cristiano Foderi, Adalid Salazar Garcia, Teresa López de Armentia, Elena Bresci, Fabio Salbitano

**Affiliations:** 1 Department of Agriculture, Food, Environment and Forestry (DAGRI), Università degli Studi di Firenze, 50145 Firenze, Italy; francesco.cantini94@gmail.com (F.C.); cristiano.foderi@unifi.it (C.F.); elena.bresci@unifi.it (E.B.); fabio.salbitano@unifi.it (F.S.); 2 Instituto de Capacitación del Oriente (ICO), Vallegrande, S/N, Bolivia; direccion@ico-bo.org; 3 Asociacion Zabalketa, Getxo, 48930 Bizkaia, Spain; teresa@zabalketa.org

**Keywords:** sustainable land and water management, remote sensing, GIS, agricultural intensification, water pollution, precipitation shift, CHIRPS, integrated hazard assessment, deforestation

## Abstract

The “Valles Cruceños” rural region plays a fundamental role for securing food and other resources for the neighboring, and fast sprawling, city of Santa Cruz de la Sierra (Bolivia). Due to the increasing pressure on its natural resources, the region is affected by progressive and severe environmental degradation, as many other rural regions in South and Central America. In this situation, sound policies and governance for sustainable land management are weak and not supported by data and scientific research outputs. With the present study, we aim at developing a novel and practical integrated hazard analysis methodology, supporting the evidence-based understanding of hazard patterns and informing risk assessment processes in the urban-rural continuum. Firstly, the main environmental hazards affecting the area were identified via questionnaire campaigns, held by the staff of local municipalities. Focusing on the hazards mostly perceived by the inhabitants of the region, including deforestation, water pollution and precipitation changes, hazard maps were created by using multiple environmental hazards indicators. An integrated hazard map was then built in a GIS environment, after a pair-wise comparison process. The maps represent a first baseline for the analysis of the present status of natural resources in “Valles Cruceños” area, and the proposed approach can be scaled up for integrated environmental hazards analysis in similar areas of Latin America.

## 1. Introduction

Anthropogenic environmental hazards represent an issue of major concern in Latin America as related to the potential effects of climate change on human activities and vice-versa. Regarding land use changes, about 35% of the global lands are used for agriculture, grazing, or are urban areas. Thus, significant agriculture expansion can only take place over the tropical forests of South America, Africa and Southeast Asia, and over the boreal forests of Canada and Russia [1]. In South America, and, in particular, in the Amazon basin, in the last 40 years the deforestation occurring in tropical forests has reached an area of 650,000 km^2^ (equal to 18% of the total forested area), due to the expansion of agriculture, mainly for soybean (*Glycine max*) plantations [2,3]. Field observations [4] and modelling [5] revealed that large scale deforestation in Amazonia could alter the regional climate significantly. Indeed, the abrupt changes in vegetation cover due to deforestation reduce evapotranspiration, key for climate regulation thanks to water vapor fluxes into the atmosphere throughout the year [6,7]. Deforestation, forest fragmentation, and the associated increase in temperature and changes in dry period regimes, as well as the systematic use of fire in agriculture, alter the hydrological cycle at multiple scales. These factors are largely recurrent in the entire Amazon River watershed, comprehending both the Amazon forest portion as well as the pre-Andean biota. They produce effects on regional and local climate, impacting on ecosystem health and dynamics, while influencing drastically the access to natural resources by the human communities. Moreover, they determine dramatic changes in cultivation opportunities and can induce substantial alteration of socio-economic security. [8].

The concern about environmental issues in Bolivia is relatively new (1990s). The country does not have a system of standards for evaluating the magnitude of environmental externalities, while monitoring programs have been established very recently, allowing only an approximate quantification of the most important pollutants as well as their residues [9]. Bolivia has a high potential of environmental hazards due to its geographical, ecological, and socio–economic characteristics. It has a vast geographical and biological diversity, from a high plateau (altiplano), placed at more than 3500 m a.s.l., to valleys at mid-altitude and tropical plains [10], with some areas of the country at risk of flooding, while other parts at risk of drought [11,12]. Furthermore, Bolivia is one of the poorest countries in Latin America, with a 38% poverty rate in 2013 [13]. The problems and uncertainty, caused by the changing patterns of weather extreme events, related to climate change, are inevitably impacting the low-income component of the rural and periurban population. First of all, these latter sectors of the society have not very often a structured knowledge and economic means to prevent the effects of these events; secondly, they have rarely sufficient capacity and economic resources to react after the occurrence of such adversities; and finally, poverty conditions (as poor housing, livelihood, shortage in basic resources, absence of savings) can amplify weather extremes and climate change impacts, leading to a progressive increase of chronic poverty.

Among others, the major environmental problems that are afflicting Bolivia are:

- indoor and outdoor air pollution;
- water pollution from industries (particularly mining) and associated adverse health and ecological impacts;
- vulnerability to natural disasters and climate change risks;
- inadequate solid waste management;
- expansion of the agricultural frontier and unsustainable land use, resulting in deforestation, loss of biodiversity, and soil erosion.

Most of these problems have direct economic and social impacts, such as the loss of agricultural productivity and an increase in water treatment costs, induced by soil erosion and water pollution, while others have high health costs. Since two thirds of Bolivians already live in urban areas, where the population is growing at a rate six times higher than in rural areas since 2000 (2.4% versus 0.4% in 2009), urban environmental issues are likely to become increasingly important for the environment and the overall development agenda of Bolivia [14].

In the present situation, tools for evidence-based understanding of hazard patterns should be developed, in order to inform and support sound policies and governance for a sustainable resources management, both at the local and at the national scale. 

In the framework of environmental analysis, risk can be defined as a function of three elements [15,16]:

(1) hazard occurrence, namely the frequency of returning period at a given magnitude of certain hazardous event;
(2) element at risk, namely the value of the elements exposed at the consequence of the given hazardous event;
(3) vulnerability, namely “the degree of loss to each element should a hazard of a given severity occur” [17].

United Nations [18] define a ‘hazard’ broadly as “a potentially damaging physical event, phenomenon or human activity that may cause the loss of life or injury, property damage, social and economic disruption or environmental degradation”. In the present work, when assessing the “hazard” element of the risk function, we adopted a simplified approach, by mapping suitable “hazards indicators”, namely values representative of each hazard occurrence probability. This approach was justified by the need of adopting a methodology suitable for a data-scarce area, as well as developing hazard indicators map to be delivered to local administrations. Similar example of hazard indicators mapping can be found in [19,20,21].

The aim of the research is to define and test a procedure of generating integrated hazard maps based on the spatialization of multiple hazards indicators. The research approach, based on the conjunctive utilization of a participatory questionnaire and GIS-based weighting of different hazard indicators, will serve to support the decision makers in managing the increasing impacts of productive agriculture in the area, while adopting integrated planning management tools towards sustainability and risk reduction.

The approach is tested for the so-called “Valles Cruceños” region, i.e., a mountain system of the western part of the Department of Santa Cruz, located at 18°16′ S–64°0′ W. The area is a remarkable hotspot of many urban-rural dynamics, potentially harmful for environmental health, since it produces food for a large part of the population of the city of Santa Cruz de la Sierra (2.6 million residents). This latter one is the leading Bolivia’s city (and the second in Latin America) for population growth and soil sealing, with an estimated average annual growth rate of 3.98% between 2006 and 2020 [22]. Because of the fast growth of the city population, there is a tremendous increase in food demand, requiring the continuous expansion of cultivations and rangelands [23]. Highly related to these issues, the area is experiencing problems of water pollution, while modifications of precipitation patterns are causing alarming conditions of drought.

The proposed procedure was conceived for being fully replicable and can be scaled up for integrated environmental hazards analysis in similar areas of Latin America, posed under pressure due to the increasing resources demand, given by urbanized populations. The research was realized in the framework of the project “Diagnostico socioambiental, riesgos ambientales y grupos vulnerables en los Valles Cruceños”, funded by NGO Instituto de Capacitación del Oriente (ICO).

## 2. Materials and Methods

### 2.1. Study Area: Los Valles Cruceños

**Figure 1:**
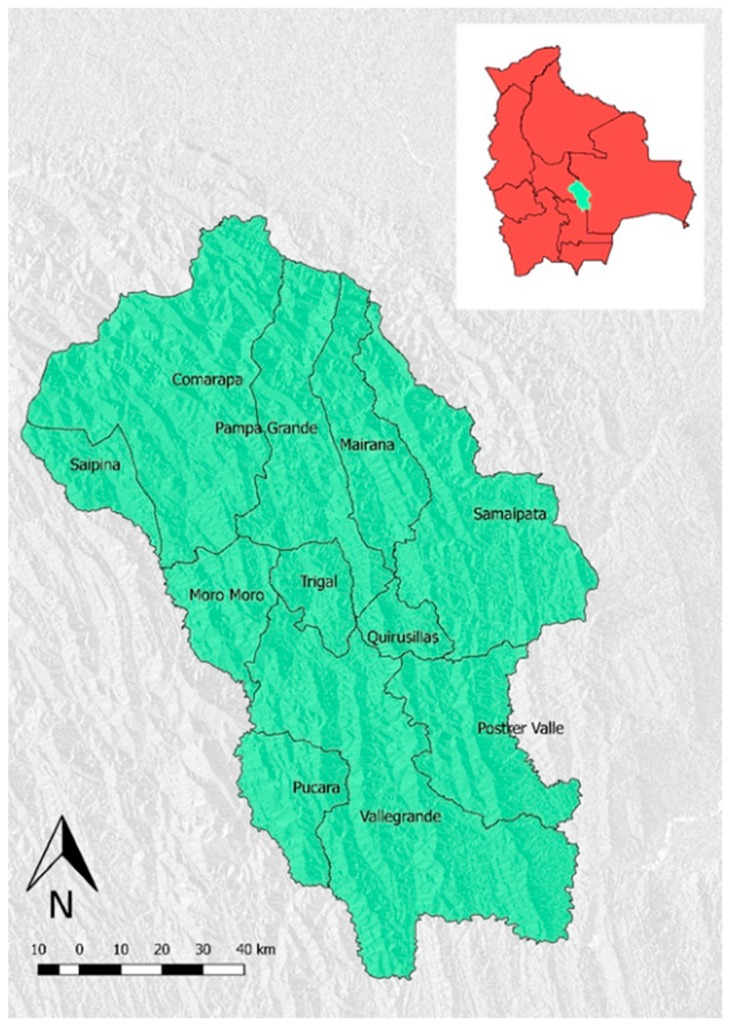
Study area and municipalities of “Valles Cruceños”, Santa Cruz department, Bolivia.

The “Valles Cruceños” region is located in Southern Bolivia, within the Department of Santa Cruz de la Sierra, bordering the Departments of Cochabamba and Chuquisaca. The region consists of three provinces (Florida, Manuel Maria Caballero and Vallegrande) including eleven municipalities: Comarapa, Mairana, Moro Moro, Pampa Grande, Postervalle, Pucarà, Quirisillas, Saipina, Samaipata, Trigal and Vallegrande (Figure 1). It is one of the poorest and least developed areas of the Department. In this context, the main land uses are agriculture and rangelands [24]. 

The study area covers 1,382,705 ha and has a population of 82,727 inhabitants [20], the most employed in small to large-scale agriculture and pastoralism. It is characterized by a wide morphological and landscape diversity, from the high mountains (3300 m a.s.l.) of the Sub-Andean zone of the “Cordillera Oriental de Los Andes”, to the low mountains, until the “Tierras Bajas” (460 m a.s.l). Among all the ecoregions present in Bolivia, the “Valles Cruceños” region contains Tucuman-Bolivian forest, Inter-Andean Dry forest, Chaco Serrano and Yungas forest [25]. The climate can be described as temperate in the eastern and subtropical part, while in the western part is characterized by a high fragmentation of ecosystems and habitats, ranging from very humid forests to dry forests [26]. Precipitation patterns in the area are extremely variable at close distances, ranging from 400 mm to 2000 mm in few kilometers. The rainy period is from December to March, while the rest of the year is mostly dry.

### 2.2. Participatory Analysis of Community Perception of Environmental Hazards

Prior to the geographical data collection and evaluation, the identification and the ranking of environmental hazards in the area of the municipalities of “Valles Cruceños” were carried out based on questionnaires, collected in meetings held with the stakeholders of the communities of Pucara, Pampagrande, Quirussilas, Postrervalle and Comarapa. The evaluation was based on a hazard scale from 0 to 50, with a score calculated according to the criteria shown in Appendix A. According to the analysis, deforestation, water pollution and precipitation shift hazards were selected.

### 2.3. Environmental Hazard Indicators

For the present work, the definition of risk (hazard) indicator as reported by the Deutsche Gesellschaft für Internationale Zusammenarbeit (GIZ) GmbH [27,28] was adopted: “(A good indicator) is clear in its direction, i.e., an increase in value is unambiguously positive or negative with relation to the factor and risk (hazard) component”.

#### 2.3.1. Deforestation Hazard (Hd) 

“Valles Cruceños” are prone to an increasing rate of deforestation. The area, affected by the expansion of the interface among urbanized areas, located around the city of Santa Cruz de la Sierra, is subject to a growing phenomenon of conversion of the land use, from forest to agricultural land, with a deforestation estimated at up to 60% in 2050, which represents an annual deforestation rate of 3.7% [29]. Previous estimates, more conservative, indicate an equal rate of deforestation to approximately 0.9% [30]. 

In order to generate a deforestation hazard indicator (Hd) map, data were processed through QGIS (QGIS.ORG, Zurich, Switzerland) and GRASS GIS (Open Source Geospatial Foundation, Beaverton, OR, USA) software. The analysis has been based on the Global Forest Change (GFC) database, published in 2013 by Hansen et al. [31]. GFC is updated yearly and provides raster maps of tree cover percentage of the year 2000, tree cover loss and gain for each successive year, with a spatial resolution of 30 m, based on Landsat data. Cover loss and gain are binary datasets, with pixel value of one if deforestation/afforestation has occurred, and 0 if not.

**Figure 2:**
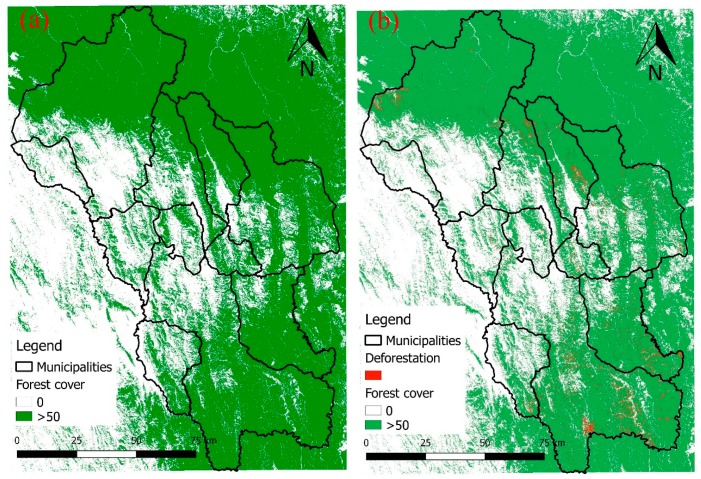
Forest cover changes in the last two decades [31]. (**a**) Forest cover at the year 2000. (**b**) Forest loss between the years 2000–2017. Maps were created considering only pixels with a value of forest >50% as “forested”.

Hd map analysis was referred to the period 2000–2017 using maps of tree cover extent (Figure 2a) and tree cover loss (Figure 2b). The assessment of deforestation hazard was limited to areas with tree cover greater the threshold of 50% at 2000 and the tree cover loss areas at 2017 to validate the model.

For the creation of the Hd map, some factors, strictly linked to land use changes, were identified as proxy variables to model the deforestation hazard, calculated starting from both GFC database and geographic map layers, available from the national geographic data portal (GEOBOLIVIA) (Table 1).

The probability of a forest area to be deforested was assumed greater in proximity of some physical elements of the landscape. A descending rescale was applied to the distance raster calculated from each element, in order to assign higher values to the nearest distance (where the hazard is assumed to be greater). The rescale classes are reported in Table 2. Each pixel of the map was reclassified according to its distance to water bodies (water bodies distance factor, Fw), agricultural areas (Fa), breeding areas (Fb), population centers (Fp) and deforested areas at the year 2000 (Fd). Additionally, a slope factor was calculated according to the terrain slope (Fs), assumed non influent over a value of 60% that has been considered the limit slope for agricultural purposes. The factor maps were used to generate a weighted sum of the resulting raster maps. Since not all the elements have the same importance, it was established to derive the relative priorities (weights) for each map. The comparison matrix adopted is shown in Table 3. Cells in comparison matrices have a value from the numeric scale shown in Table 4 to reflect the relative preference (also called intensity judgment or simply judgment) in each of the compared pairs.

Weighted values (W) were calculated as the average value of each row of the normalized matrix (Table 5).

The Hd map was generated by Equation (1):

(1) $$Hd^{\prime }=FaWa+FbWb+FwWw+FrWr+FsWs+FdWd.$$

This map was calculated for all the study areas; in order to have only the map of the forest areas, we used the operation:

(2) $$Hd=Hd^{\prime }\;x\;ForestCover$$

considering only the pixels with forest cover >50%.

#### 2.3.2. Deforestation Hazard Validation

In order to validate the deforestation hazard data model, a comparison between the Hd map pixels and the GFC cover loss dataset at 2017 was performed.

#### 2.3.3. Water Pollution Hazard (Hw)

In order to have a spatialized information on the water pollution hazard throughout the territory of the “Valles Cruceños”, a specific water pollution hazard (Hw) indicator was defined by using the information about the farming and breeding activities, provided by the agro–economic census [32], reporting the most updated data at the time of the analysis. This allowed the identification of the percentage of agricultural or grazing surfaces (A) of each municipality. These latter were calculated as the ratio between the sum of agricultural and grazing areas, and the total area of each municipality. To the municipalities with the most extensive surfaces was given a hazard index of 4, to those with slightly inferior surfaces 3 and so on up to 1 (Table 6).

To further evaluate the state of pollution caused by farming activities, a detailed water pollution analysis was carried out by sampling different water sources in the region. As a consequence of the great extension of the territory of the “Valles Cruceños” and of the elevated cost of each analysis, it was established to execute a pilot analysis in order to give a baseline information on the pollution state of the waterbodies of the study area. To establish the areas where the water quality analysis of the pilot sampling was to be carried out, it was concluded to use the information about the farming activities provided by [32] (Table 7 and Table 8).

As shown in Table 7 and Table 8, the municipalities with the highest agricultural area percentage were Mairana, Trigal, Pucarà and Pampa Grande, while the ones with the highest pastoral surface percentage were Pucarà, Moro Moro and Postervalle. Pucarà municipality was found to have both a high percentage of agricultural and grazing area, but no sampling has been carried out there because of the absence of useful wells for the analysis. 

**Figure 3:**
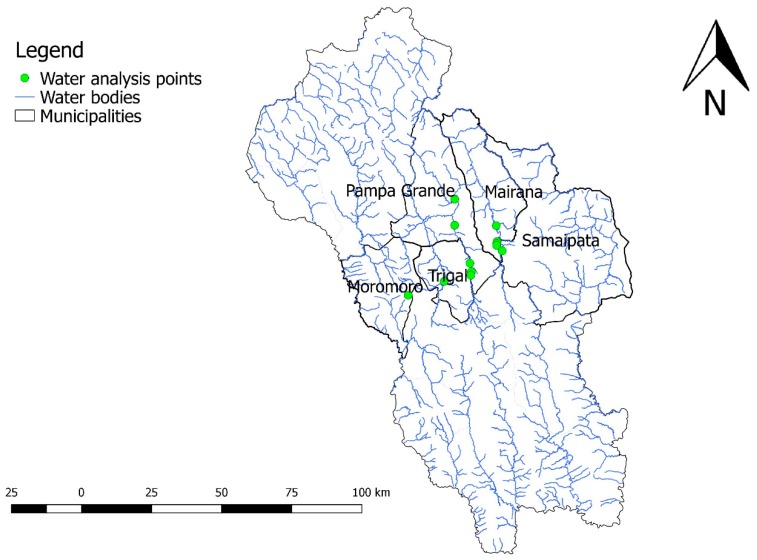
Abstraction points for water samples, “Valles Cruceños” region, Bolivia, 2019.

Water sampling for the analyses were carried out in the municipalities of Moro Moro, Pampa Grande, Trigal, Mairana and Samaipata. The list of the water points sampled is shown in Table 9, while locations are shown in Figure 3.

For each location, two samples of water were taken by the means of two different containers. The first one consisted of a sterilized plastic bag with a maximum capacity of 200 mL, mainly used for microbiological analyses while the other, used for chemical ones, was a simple plastic bottle with a capacity of 500 mL. Before carrying out any sampling operation, it was necessary to sterilize the metal sampler used for the withdrawal of water. This operation was carried out by burning a small amount of ethyl alcohol which was placed each time in the sampler. 

Analysis for pH, electrical conductivity, turbidity, nitrite, nitrate, temperature, total coliforms fecal coliforms (by using the 200 mL container), glyphosate and ammonia nitrogen (the last two analyzed on the samples taken in the 500 mL container) were realized on the collected water samples. Samples stored in the 200 mL container were withdrawn and delivered to the laboratory within 24 hours, required to guarantee representative results, respecting the requirements of transportation in stable temperature conditions. Glyphosate analyses were added upon specific request of the Gobierno Autónomo Departamental de Santa Cruz. This latter component represents a widely used herbicide, reported to induce various toxic effects in non-target species. Recently, some studies have found it to be carcinogenic for humans [33,34].

The values of the parameters were benchmarked with the respective limits, posed by Bolivian Law for drinking water.

#### 2.3.4. Precipitation Shift Hazard (Hp)

The precipitation shift hazard (Hp) was not inserted in the questionnaires used for the participatory analysis. However, the potential threat related to a change in precipitation patterns due to climate change emerged from most of the informal discussions held with the environmental experts, met during the project. Moreover, since most of the wealth of this area comes from farming and grazing activities, a change in the rainy regime could lead to water imbalances that would affect the quantity and/or quality of the crops produced.

Among other approaches, drought indices analysis represents a valid framework to monitor and to assess drought occurrence, as well as variation of rainfall and drought patterns [35,36]. For the present study, the standardized precipitation index (SPI), proposed by McKee et al. [37] was adopted. It is widely used for drought forecasting and can be used in risk assessment and decision-making, being a standardized indicator based on probabilistic analysis [38]. SPI is calculated using the long-term monthly rainfall record at a specific location [39]; the long-term record is then adapted to a probability distribution, which is transformed into a normal distribution. Positive SPI values indicate a rainfall increase compared to the median precipitation and negative SPI values indicate a rainfall decrease.

For this study, the monthly precipitation data retrieved from Climate Hazards Group Infrared Precipitation with Stations (CHIRPS) database [40], from 1981 to 2017 were analyzed. CHIRPS is a new quasi-global (50° S–50° N), high resolution (0.05°), daily, pentadal and monthly precipitation dataset, composed of raster images of accumulated precipitation values, distributed in 500 x 500 m cells. It is built on a long period of record precipitation estimates, based on infrared cold cloud duration (CCD) observations [40]. The algorithm for rainfall estimation is built around a 0.05° climatology, that uses satellite information to represent poorly calibrated positions and covers the period from 1981 onwards. 

For each year, a three-month SPI was calculated based on CHIRPS time series, considering the period of rainy season, from January to March. This method was used to compare the precipitation over a specific three-month period with the total precipitations from the same three-month period for all the years of the record, evaluating inter annual anomalies of precipitation. With a linear interpolation process, the values of the SPI trends were calculated. For negative trends, the Mann–Kendall statistical test was performed to identify which trends had a 95% of statistical significance. 

Root mean squared error (RMSE) and RMSE-observations standard deviation ratio (RSR) were utilized for a validation of the CHIRPS database of the study area, by comparing monthly rainfall time series [41]. Both RMSE and RSR value of 0 indicate a perfect fit. According to Moriasi et al. [42] (Moriasi et al., 2007 [42] refers to discharge time series analysis. However we considered their reference as a standard for RSR.) RSR values below 0.5 indicate a “very good” value of correlation, RSR between 0.5 and 0.6 indicate a “good” correlation, while, with RSR between 0.6 and 0.7, this latter one is considered “satisfactory”. RSR values above 0.7 are typically indicating “unsatisfactory” values of correlation.

Results are reported in Table 10. The weather stations of Comarapa, Mataral and San Juan del Potrero show a “satisfactory” result, while Yerba Buena station shows an “unsatisfactory” value of RSR. Vallegrande airport station is found to have an RSR index of 0.5. However, this latter result is driven by the fact that Vallegrande is one of the Bolivian stations used for generating the CHIRPS dataset [40]. Stations data were retrieved by SEARPI—Servicio de Encauzamiento de Aguas y Regularización del Río Piraí, Bolivia.

Given the limited availability of data for the area of study and based on the overall acceptable results for the stations selected for validation, we decided to consider the CHIRPS dataset as appropriate for the analysis.

Table 11 shows the hazard values that have been assigned to the SPI classes. The map was produced for a 0.05° cell raster grid representative of “Valles Cruceños”.

#### 2.3.5. Integrated Environmental Hazard Mapping

In order to have a complete overview on the environmental hazard of “Valles Cruceños” region, it was established to perform an integrated hazard map using the information obtained by the hazard maps previously investigated. The integrated hazard map was the weighted sum of the map of Hd, Hw and Hp. The coefficients adopted to weight the variables in the final equation were obtained from the comparison matrix reported in Table 12. Cells in comparison matrices had a value from the numeric scale shown in Table 4 to reflect the relative preference in each of the compared pairs. Normalized comparison matrix is show in Table 13.

For each pixel mapped, an integrated hazard indicator (H), was calculated as follows:

(3) H=0.6Hd+0.2Hw+0.2Hp

## 3. Results

### 3.1. Community Perception of Environmental Hazards

For the analysis of the main hazards present in the Valles Cruceños area, the phenomena with a hazard score over 30 on average on the municipalities were considered, adding also the ones with an impact value equal to or greater than 30 in one or more individual municipalities. For each hazard analyzed, a correspondent indicator was assigned (Appendix B).

#### 3.1.1. Validation of Deforestation Hazard Map Results

As shown in Table 14, it is possible to affirm that the hazard map reports quite accurately the information on deforestation occurrence and can then be used for future deforestation hazard evaluation. In fact, class 3, correspondent to medium/high hazard, comprehends about the 75% of already deforested areas. This means that from the year 2000 until the year 2017 about 75% of the areas that were deforested were characterized by a medium/high hazard value. 

#### 3.1.2. Deforestation Hazard Map

**Figure 4:**
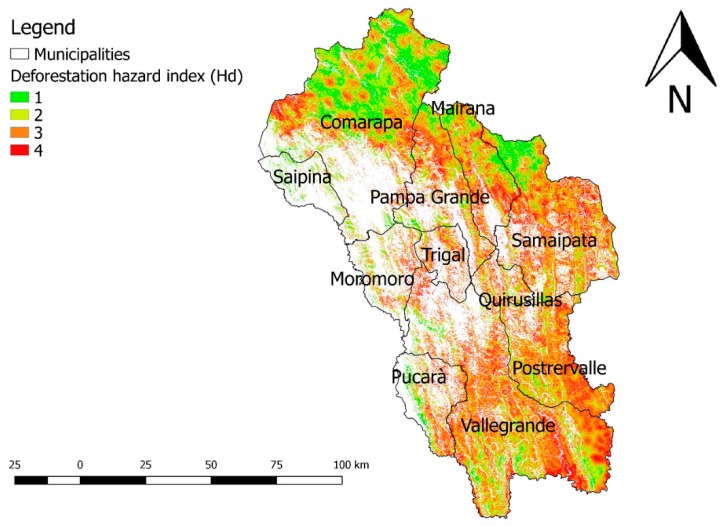
Deforestation hazard map (Hd) of the “Valles Cruceños” region. Hd varies from 1 (lower) to 4 (higher). Bolivia, 2019.

The map of Hd values the study area is shown in Figure 4.

Hd varies from 1 (low) to 4 (high). The areas with the largest surfaces of high deforestation hazard are in the South of the study region, in the municipalities of Vallegrande and Postervalle. The result can be explained with the characteristics of the area, with many flat areas and big rivers. The second major hot spot is located in the northern part of the region, in the Amborò National Park, including the municipalities of Comarapa, Pampa Grande, Mairana and Samaipata. 

### 3.2. Water Pollution Hazard Results

#### 3.2.1. Water Pollution Hazard Map

**Figure 5:**
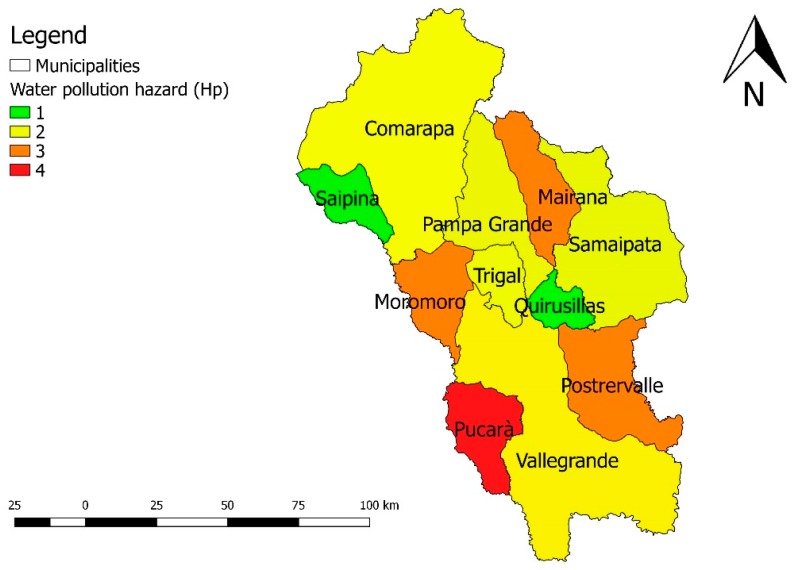
Water pollution hazard (Hw) map of the “Valles Cruceños” region, Hw varies from 1 (lower) to 4 (higher). Bolivia, 2019.

The results of the analysis of Hw hazard indicator, expressed at municipal level, are shown in Table 15 and Figure 5.

The municipalities that present the highest hazards of water contamination are: Pucarà, Moromoro, Postervalle and Mairana. 

#### 3.2.2. Water Quality Analysis

The results of the water quality analyses are shown in Table 16, where the requirements of the Bolivian Water Law (both for drinking and for irrigation purposes) are reported in the last row. 

As shown in Table 16, results of the water analysis should be analyzed depending on the water use. In all sampling points, water is characterized by an overall good quality status when considering the irrigation use, while the limits imposed by the “Norma Boliviana NB-512” for drinking purposes are not matched due to a high concentration of Coliforms. This latter factor represents an indicator relevant to the impact on breeding activities on natural resources [43], establishing the need to take actions to improve water quality. In the framework of the project “Diagnostico socioambiental, riesgos ambientales y grupos vulnerables en los Valles Cruceños”, the following actions were then suggested:

- reduce the incorporation of bacterial contamination to water bodies, mainly due to cattle feces, isolating the slopes of the area where this economic activity takes place.
- implement methods of elimination of bacterial contamination, through filter systems and/or chemical disinfection, such as chlorination.

### 3.3. Precipitation Shift Hazard Results

**Figure 6:**
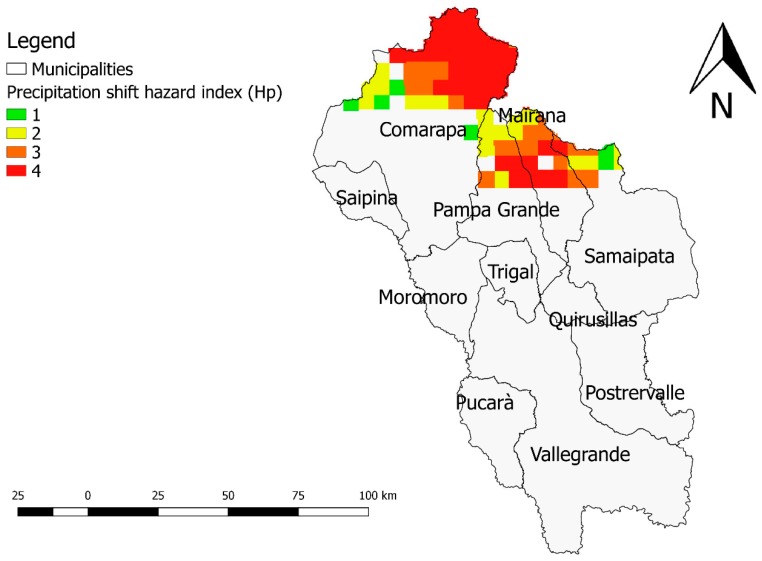
Precipitation shift hazard map (Hp) of the “Valles Cruceños” region, Hp varies from 1 (lower) to 4 (higher). Bolivia, 2019.

The map of Hp values is shown in Figure 6.

Elevated Hp shift values were found in the northern part of the “Valles Cruceños” region where the Amborò National Park (Figure 6) is located. The main vegetation system of this area is the humid sub-tropical forest and the climate is characterized by heavy rainfall with an average temperature of 24 °C [44]. 

### 3.4. Integrated Environmental Results

**Figure 7:**
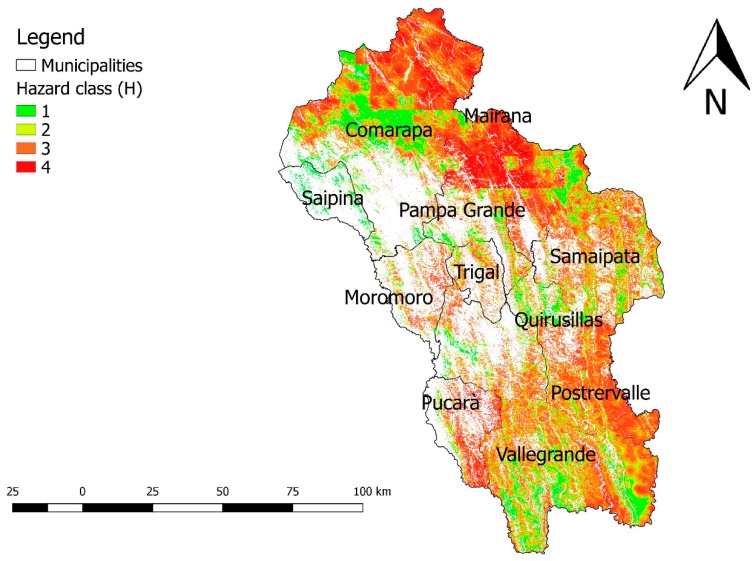
Integrated hazard (H) map of the “Valles cruceños” region, H varies from 1 (lower) to 4 (higher). Bolivia, 2019.

The map of H values is shown in Figure 7.

The “integrated hazard map”, representing the values of the H indicator for integrated hazard, allows an overview of all the elements of hazard that afflict the study area. The results show that the northern part of the region was the one with the highest hazard values. This is mainly due to the sum of the deforestation and drought hazard maps. The municipalities of Trigal and Pucarà, which in the maps of drought and deforestation had values close to zero, in the integrated hazard map, were found to have higher values of environmental hazard due to the water pollution hazard map. The municipalities of Vallegrande and Postervalle highlight a high environmental hazard that is mainly due to the hazard of deforestation. 

## 4. Discussion

The “Valles Cruceños” region presents a high geographical and socio-economic diversity in space and time, exposing lands and communities to a wide range of environmental hazards. The tropical plains are critically exposed to both deforestation and floods, and much less to erosion. Mountain areas have a lower risk of deforestation but the risk of erosion is very high [11] whenever land cover changes drastically exposing slopes to erosion and soil fertility loss. Referring to the socio-economic structure of the region, the strong rural character and the economic prevalence of agricultural and livestock activities, make the community highly vulnerable to environmental perturbations, such as a prolonged period of drought that would drastically decrease crop yields. The consequences of such events could affect dramatically the primary sources of income of a larger part of the community while indirectly damaging the whole economy of the valleys [24]. 

A change in the precipitation regime could lead to catastrophic consequences since vegetation is not prepared to deal with long periods of drought and it could become highly susceptible to fires. The further critical aspect refers to the low adaptability to wildfires shown by the vegetation types currently present in the region, particularly in “Bosque Tucumano-Boliviano” and the “Yungas”. Deforestation in these two types of vegetation leads to serious environmental problems, such as the loss of biodiversity and the reduction of the water regulation function as well as soil erosion. 

If deforestation and drought are considered separately, they express a type of information that does not take into account the intimate and reciprocal interaction of the two hazards. For example, problems connected with deforestation combined with drought problems can lead to serious consequences such as the occurrence of large fires, in fact in a situation of absence of water in the soil due to a prolonged period without rain combined with the deforestation technique called “slash and burn” can led to the occurrence of large fires that are difficult to control. This is what happened with the large forest fire that took place in northern Amazonia for two months during the year 1998. It was associated to a very long period of drought during the 1997–1998 and it is likely to have been the largest forest fire of modern times in Amazonia [45]. This technique is not only associated with the hazard of fires but also causes serious problems with regard to soil erosion. 

On the basis of the results of the present study, it is possible to state that the “Valles Cruceños” represents a theatre for various types of environmental hazards and the local administrations and decision makers must tackle the potential risks through a sound environmental governance. Integrated mapping, as proposed in the present paper, looks as a promising tool to support decisions and contribute to contrast some of the dramatic effects of environmental hazards. Due to the currently available data, the study has some limitations, above all concerning the harmonization of scales in mapping. In fact, the integrated hazard map was created by multiplying the Hd, Hp and Hw maps which have different pixel dimensions (Hd: 30 x 30m, Hp: 500 x 500m, Hw: the entire municipalities). Other limitations concern scaling down the approach to a greater detail. Due to the type of research and the allocated budget, it was not possible to collect water samples in all municipalities involved in the project because of the high price of each analysis. Although the method generated sound results, the validation of the Hd map can be considered acceptably good, but not excellent. 

## 5. Conclusions

The present work aimed at providing a simple integrated framework for multiple environmental hazard analysis, based on the concept of hazard indicators. The approach is intended to serve as a support tool for informing sound environmental policies in those entire context where an healthy environment is posed at risk by an increasing land resources demand, given by a neighboring urbanization. The rural−urban dynamics occurring in the area of “Valles Cruceños”, in Bolivia, posed under heavy pressure by the city of Santa Cruz de la Sierra, resulted to be an informative case study in this sense. Here, deforestation, water pollution and precipitation shift hazards were considered the most dangerous and adequately mapped, with an evidence-based approach.

Deforestation represents one of the major environmental concerns not only in Bolivia but in the entire Amazonian basin. In the framework of the analysis carried out in the study area, the municipalities showing the highest values of deforestation hazard are those situated in the Southern part of the study area (Vallegrande and Postervalle). This result can be referred to the morphology of the zone: the lowest altitudes and the high recurrence of flat areas and rivers, led to a higher agricultural and grazing pressure in the last decades as the demand of food by the increasing population of Santa Cruz de la Sierra grew. Another “hot spot” is located in the north part of the region, in the Amborò National Park area (municipalities of Comarapa, Pampa Grande, Mairana and Samaipata). The two areas have similar vegetation, characterized by the presence of a dense evergreen forest. Deforestation in these conditions leads to serious environmental problems such as the loss of biodiversity and the reduction of the water regulation function as well as to soil erosion. Increasing controls in areas with a higher deforestation hazard is needed in order to try to limit this phenomenon. 

Moreover, in addition to the issue related to forest cover loss, extension of agricultural frontiers could determine a potential source of environmental risk at different level. Even if subsistence agriculture continues to play an important role in the dynamic of deforestation, medium and large size farms, mainly specialized in sugarcane, soybean and cotton, could become a future environmental hazard factor also in terms of water and soil quality due to the growing use of agro−chemicals and pesticides.

Concerning the water analysis, it is possible to state that in the study area there were not problems of water chemical pollution; but, due to the increasingly massive use of herbicides and chemical fertilizers in agriculture, it is advisable to monitor continuously the health status of the waters. However, water analyses highlighted the presence of Coliforms, which can create problems when water is consumed for domestic use. In order to limit the presence of Coliforms, largely due to grazing, it becomes decisive to integrate the management of livestock at landscape level so to implement filtering green infrastructure components in most densely grazed zones. Hw map can be then considered an important tool to plan a future monitoring scheme of the water quality because it allows to produce a dataset on which municipalities can get easily a communicative feedback on where most likely polluted water is. 

The last factor that has been investigated is the hazard of precipitation shift. Since it is a direct consequence of the climate change, at the level of the “Valles Cruceños” region it is not possible to carry out actions able to interrupt the hazard of drought. The only actions that can be done are to try to mitigate the impacts of drought on the population and on the environment of the study area. For example, a long period of drought increases the hazard of fire, especially in tropical forest areas which have always had high soil moisture levels. To limit the hazard of fire, all those operations that include the use of fire, among which the deforestation technique “slash and burn”, should be avoided.

The integrated hazard map, produced as last point of the analysis, can allow the evaluation of all the hazards that are found in the study area and their connections. Nevertheless, it should be observed that the map was produced with datasets at different resolution, especially considering the water pollution hazard, clustered at municipality level, limiting the potential of its application for very small-scale analyses. Due to this, the complementary use of integrated and single hazard map is recommended for having a sounder overview of environmental issues detected.

In addition to this, it should be noticed how the process can be easily repeated once a new version of Hd, Hp or Hw map will be produced. In this regard, further monitoring and data generation about water pollution analysis is recommended as a first improvement of the process, as well as further refinement of Hd map. However, at the current state of the analysis, all the presented maps can be easily consulted and interpreted, in order to have a baseline information to inform environmental management in the various municipalities of the “Valles Cruceños” region.

The proposed approach was implemented in the framework of the project “Diagnostico socioambiental, riesgos ambientales y grupos vulnerables en los Valles Cruceños”, involving also a large part of the local administrations. This latter characteristics, as well as the emblematic case study for which the process was tested, allow the proposition of the present approach for being tested and scaled up in other similar contexts of South and Central America.

## Figures and Tables

**Table 1:** Description of data collected and processed for the study area.

Name	Data Type	Description	Source
Forest cover 2000	Raster (geotiff)	Forest cover map of the year 2000	Hansen et al., 2013 [31]
Deforestation (2000–2017)	Raster (geotiff)	Deforestation map of the period 2000–2017	Hansen et al., 2013 [31]
Deforestation (2000)	Raster (geotiff)	Deforestation map of the year 2000	Hansen et al., 2013 [31]
DEM	Raster (geotiff)	Digital Elevation Model of the “Valles cruceños” region	GEOBOLIVIA
Water bodies	Vector (shapefile)	Total hydrographic pattern	GEOBOLIVIA
Population centers	Vector (shapefile)	Map of the population centers points	Elaboration of ICO
Roads	Vector (shapefile)	Map of the roads of the study area	GEOBOLIVIA
Agricultural areas	Vector (shapefile)	Map of the agricultural areas of the study area	GEOBOLIVIA
Breeding areas	Vector (shapefile)	Map of the breeding areas of the study area	GEOBOLIVIA
Slope	Raster (geotiff)	Map of the slope	Elaboration from DEM

**Table 2:** Hazard values of slope factor (Fs), factor of proximity to water bodies (Fw), factor of proximity to roads (Fr), population centers (Fp), agricultural areas (Fa) and breeding areas (Fb) and factor of proximity to areas deforested in 2000 (Fd). The classification for Fr, Fo, Fa and Fb is the same.

**Slope (%)**	**Fs**	**Distance from water bodies (m)**	**Fw**
0–20	4	0–100	1
20.1–40	3	100.1–300	4
40.1–50	2	300.1–600	3
50.1–60	1	600.1–1000	2
60.1–max	0	1000.1–max	1
**Distance from roads, population centers, agricultural areas and breeding areas (m)**	**Fr, Fp, Fa, Fb**	**Distance from areas deforested in 2000 (m)**	**Fd**
0–500	4	0–500	4
500.1–2000	3	500.1–1000	3
2000.1–5000	2	1000.1–2000	2
5000.1–max	1	2000.1–max	1

**Table 3:** Comparison matrix of the criteria for the determination of the deforestation hazards.

Landscape Element	Agricultural Areas	Breeding Areas	Water Bodies	Roads	Population Centers	Slope	Deforestation
**Agricultural areas (a)**	1	1	1	3	3	0.33	0.33
**Breeding areas (b)**	1	1	1	3	3	0.33	0.33
**Water bodies (w)**	1	1	1	1	3	0.11	0.11
**Roads (r)**	0.33	0.33	1	1	3	0.11	0.11
**Population centers (p)**	0.33	0.33	0.33	0.33	1	0.11	0.11
**Slope (s)**	3	3	9	9	9	1	1
**Deforestation (d)**	3	3	9	9	9	1	1

**Table 4:** Comparison matrix values.

Much More Important	More Important	The Same	Less Important	Much Less Important
9	3	1	1/3	1/9

**Table 5:** Normalized comparison matrix and calculation of weights for hazard indicator (Hd) map generation.

Landscape Element	Agricultural Areas	Breeding Areas	Water Bodies	Roads	Population Centers	Slope	Deforestation	Weights (W)
**Agricultural areas (a)**	0.103	0.103	0.0447	0.11	0.1	0.11	0.111	**0.098**
**Breeding areas (b)**	0.103	0.103	0.0447	0.11	0.1	0.11	0.111	**0.098**
**Water bodies (w)**	0.103	0.103	0.0447	0.04	0.1	0.04	0.037	**0.066**
**Roads (r)**	0.034	0.034	0.0447	0.04	0.1	0.04	0.037	**0.046**
**Population centers (p)**	0.034	0.034	0.0149	0.01	0.03	0.04	0.037	**0.029**
**Slope (s)**	0.31	0.31	0.4029	0.34	0.29	0.33	0.333	**0.332**
**Deforestation (d)**	0.31	0.31	0.4029	0.34	0.29	0.33	0.333	**0.332**

**Table 6:** Values of the water pollution hazard (Hw) for different percentage of agricultural and grazing areas (A), Hw varies from 1 (lower) to 4 (higher).

Percentage of Agricultural and Grazing Area (A)	Water Pollution Hazard Indicator (Hw)
<8%	1
8.1% ≤ A < 12%	2
12.1% ≤ A < 16%	3
A ≥ 16.1	4

**Table 7:** Agricultural areas in the different municipalities of the study area.

Municipality	Municipal Area (ha)	Agricultural Area (ha)	Agriculture/Land Cover Rate (%)
Samaipata	192,503	10140.5	5.27
Pampa Grande	100,661	6432.9	6.39
Mairana	74,361	7035	9.46
Quirusillas	28,716	1509.9	5.26
Comarapa	331,741	11170.1	3.37
Saipina	44,995	2326.1	5.17
Vallegrande	321,629	14106	4.39
Trigal	40,046	3127.8	7.81
Moro Moro	68,030	3602.8	5.30
Postrer Valle	111,699	3267.3	2.93
Pucará	68,324	4626.5	6.77

**Table 8:** Animal husbandry data in the different municipalities of the study area

Municipality	Pasture Surface (ha)	Pasture Surface Percentage (%)	Cattle (*N*)	Cattle/ha	Poultry Farms (*N*)	Poultry Farms/ha
Samaipata	5394.8	2.80	14,894	0.077	842895	4.379
Pampa Grande	5031.3	5.00	16,642	0.165	167807	1.667
Mairana	3259.8	4.38	8639	0.116	1,003,203	13.491
Quirusillas	474.3	1.65	2908	0.101	4940	0.172
Comarapa	19,691.9	5.94	17,790	0.054	185,337	0.559
Saipina	387.8	0.86	4070	0.090	21,708	0.482
Vallegrande	17,513.1	5.45	38,002	0.118	94,887	0.295
Trigal	1426.3	3.56	6800	0.170	31,364	0.783
Moro Moro	7038.2	10.35	6072	0.089	6703	0.099
Postrer Valle	11,355.7	10.17	16,533	0.148	10,010	0.090
Pucará	8531.9	12.49	6755	0.099	7030	0.103

**Table 9:** Locations of water points sampled.

*N*	Type	Municipalities	Site	Elevation (m a.s.l.)	Longitude (°)	Latitude (°)
1	River	Mairana	Mairana	1272	−63.9682	−18.1268
2	Well	Mairana	Bellavista	1365	−63.9657	−18.1782
3	Well	Mairana	Bellavista	1378	−63.9668	−18.1846
4	Well	Mairana	Bellavista	1382	−63.9676	−18.1902
5	Well	Samaipata	Monte Agudo	1384	−63.9487	−18.2081
6	River	Trigal	Trigal	1562	−64.1452	−18.3057
7	River	Pampa Grande	Los Negros	1237	−64.1078	−18.0409
8	Well	Pampa Grande	Basanca	1302	−64.1086	−18.1247
9	Well	Trigal	La Ramada	1432	−64.0579	−18.2475
10	Well	Pampa Grande	La Ramada	1463	−64.0530	−18.2750
11	Well	Trigal	La Raia	1469	−64.0550	−18.2865
12	River	Moro Moro	Alto Veradero	2727	−64.2668	−18.3496

**Table 10:** Values of root mean squared error (RMSE) and RMSE-observations standard deviation ratio (RSR) utilized for the validation of the Climate Hazards Group Infrared Precipitation with Stations (CHIRPS) dataset in the study area for the selected validation stations. The data from Vallegrande were used to generate the CHIRPS database value of rainfall.

Station	Lat (°)	Long (°)	Beginning	End	RMSE (mm/month)	RSR
Vallegrande	−18.2907	−64.0631	Jan-02	Dec-13	27.3	0.5
Yerba Buena	−17.5906	−64.0155	Jan-92	Dec-01	48.8	0.81
Comarapa	−17.5455	−64.3145	Jan-91	Dec-02	33.7	0.64
Mataral	−18.0755	−64.1224	Jan-87	Dec-13	36.4	0.69
San Juan del Potrero	−17.5823	−64.1719	Jan-88	Dec-09	31.2	0.68

**Table 11:** Precipitation shift hazard (Hp) values, Hp varies from 1 (lower) to 4 (higher).

Angular Coefficient SPI Value	Hazard
<−0.04	4
−0.0399 to −0.03	3
−0.0299 to −0.02	2
−0.0199 to −0.001	1

**Table 12:** Comparison matrix of the criteria for the determination of the integrated hazard.

	Hd	Hp	Hw
**Hd**	1	3	3
**Hp**	0.333333	1	1
**Hw**	0.333333	1	1

**Table 13:** Normalized comparison matrix of the criteria for the determination of the integrated hazard.

	Hd	Hp	Hw	Weights
**Hd**	0.086	0.257	0.257	0.6
**Hp**	0.029	0.086	0.086	0.2
**Hw**	0.029	0.086	0.086	0.2

**Table 14:** Hazard classes of the deforestation hazard (Hd) map of deforested areas, Hd varies from 1 (lower) to 4 (higher).

Classes	Hectares	%
1	148	0.6
2	5460	21.4
3	19,050	74.8
4	810	3.2

**Table 15:** Classification of water pollution hazard (Hw) value, Hw varies from 1 (lower) to 4 (higher).

Municipality	Agricultural Areas (%)	Breeding Areas (%)	Sum of Agricultural and Breeding Areas (%)	Hw
Samaipata	5.27	2.8	8.07	2
Pampa Grande	6.39	5	11.39	2
Mairana	9.46	4.38	13.84	3
Quirusillas	5.26	1.65	6.91	1
Comarapa	3.37	5.94	9.31	2
Saipina	5.17	0.86	6.03	1
Vallegrande	4.39	5.45	9.84	2
Trigal	7.81	3.56	11.37	2
Moro Moro	5.3	10.35	15.65	3
Postrer Valle	2.93	10.17	13.1	3
Pucará	6.77	12.49	19.26	4

**Table 16:** Results of the microbiological and chemical water analysis.

GPS	pH	Electrical Conductivity (µS/cm)	Turbidity (UNT)	Temperature (°C)	Nitrite (mg/L)	Nitrates (mg/L)	Total Coliforms (UFC/100 mL)	Fecal Coliforms (UFC/100 mL)	Ammoniacal Nitrogen NH4 (mg/L)	Glyphosate (µg/L)
1	7.77	249	37	21.4	0	0.006	80	22	<5.00	<600
2	7.32	534	22	21.1	0	0	50	8	<5.00	<600
3	7.31	574	11	20.8	0.004	0.006	45	9	<5.00	<600
4	6.94	408	18	21.4	0.006	0.001	40	7	<5.00	<600
5	7.32	7.36	12	20.5	0.008	0.01	38	7	<5.00	<600
6	7.44	841	55	21.2	0	0	70	20	<5.00	<600
7	7.1	786	52	12.5	0.0001	0.0002	68	19	<5.00	<600
8	7.75	1045	52	13.1	0.02	0.03	47	9	<5.00	<600
9	7.77	423	18	13.2	0.02	0	45	2	<5.00	<600
10	7.57	1332	11	13.5	0	0	37	2	<5.00	<600
11	7.38	1757	42	13.6	0.03	0.016	70	13	<5.00	<600
12	7.52	97	194	13.4	0.0018	0.008	65	16	<5.00	<600
**Limits**	6.5−9.0	1500	5		0.1	45	5000 (0 for potable water)	1000 (0 for potable water)	>5.00	>600

## Appendix A

**Table A1:** Disaggregation of rankings for the participatory environmental hazard evaluation proposed in Valles Cruceños region. Each impact feature (geographical extent, duration, probability, severity, reversibility) was assigned in a focus group discussion. Aggregated hazard values were calculated as the sum of each impact feature value.

Hazard Evaluation	Value
**Geographical extent**	**2–10**
REGIONAL: over-municipal dimension	10
LOCAL: Affects the concrete area and other adjacent areas.	5
PUNCTUAL: It affects the specific area of intervention but not the adjoining areas.	2
**Duration**	**2–10**
Length: greater than 10	10
Medium: from 5 to 10	5
Short: from 0 to 5	2
**Probability**	**2–10**
High	10
Moderate	5
Unlikely	2
**Severity**	**2–10**
Very serious (illegal, damage to health of people and a very high percentage of flora or fauna)	10
Severe (no significant damage to people but in flora and fauna)	7
Medium (only causes moderate and specific damage to immediate neighbors)	5
Insignificant	2
**Reversibility**	**2–10**
Irreversible: the damage is irreversible	10
High: the damage is reversible in 25 years	7
Average: the damage is reversible in 10 years	5
Low: the damage is reversible as soon as the activity is suspended	2

## Appendix B

**Table A2:** Indicators of impact and impact considered—average impact value in the four municipalities considered for the analysis.

Average Aggregated Hazard Value	Action that Generate Impact	Environmental Impact	Indicators
45	Leakage of fuels in tanks	Fire, soil contamination	Hw
42	New roads or means of communication	Settlements and expansion of agricultural borders	Hw, Hd
42	Expansion of urban sprawl	Generation of waste, loss of productive areas, and expansion of agricultural frontiers.	Hw, Hd
39	Water treatment plants	Extensive occupation of land, contamination of surface water and groundwater.	Hw
38	Plastics use (agrochemical packaging)	Contamination of water sources, soil pollution	Hw
37	Deforestation of hillsides	Landslides and erosion	Hd
35	Use of Agrochemicals	Soil pollution and environment, atmospheric, water source contamination, diseases in people and fauna, reduction of bee colonies (pollinators)	Hw
34	Deforestation	Erosion. Loss of fauna and flora, and timber species. Increase in wind erosion and nearby. Loss of regulation of temperature and rainfall. Imbalance of the local hydrological cycle	Hw
34	Slash and burn	Erosion, loss of fertile layer, deforestation, loss of water recharge surface, loss of flora and fauna	Hd
33	Grazing	Soil compaction and degradation, loss of forage species, water contamination, infiltration reduction	Hd, Hw
32	Interventions in canals	Decrease or extinction of flora and fauna.	Hd, Hw
32	Hillside cultivation	Degradation of the soil, loss of fertile layer, accumulation of sediments in the middle and lower watershed	Hd
